# Interpersonal Adaptation, Self-Efficacy, and Metacognitive Skills in Italian Adolescents with Specific Learning Disorders: A Cross-Sectional Study

**DOI:** 10.3390/ejihpe12080074

**Published:** 2022-08-10

**Authors:** Elena Commodari, Valentina Lucia La Rosa, Elisabetta Sagone, Maria Luisa Indiana

**Affiliations:** Department of Educational Sciences, University of Catania, 95124 Catania, Italy

**Keywords:** specific learning disorder, interpersonal adaptation, self-efficacy, metacognition, adolescence

## Abstract

This study aimed to explore interpersonal adaptation, generalized self-efficacy, and metacognitive skills in a sample of Italian adolescents with and without a specific learning disorder (SLD). A total of 564 secondary and high school students (males = 236; females = 328; age range: 11–19; M = 16.14, SD = 1.70) completed a set of standardized tests assessing social and interpersonal skills (non-affirmation, impulsiveness, narcissism, social preoccupation, and stress in social situations), general self-efficacy, and metacognition. Students with SLD reported a lower interpersonal adaptation than students without SLD. Furthermore, students with SLD were more impulsive and had more problems handling social situations. They also reported lower levels of self-efficacy but higher metacognition scores than peers without SLD. The use of compensatory tools was associated with better interpersonal skills and higher levels of self-efficacy in students with SLD. Finally, using these instruments is predictive of high levels of metacognitive skills in adolescents with SLD. In line with the previous literature, this study showed the presence of a gap between adolescents with and without an SLD in terms of interpersonal adaptation, general self-efficacy, and metacognitive skills in the school context. Further studies are needed on the psychological well-being of adolescents with SLD and especially on the protective role of personal, social, and environmental characteristics.

## 1. Introduction

Specific learning disorder (SLD) is among the most frequently diagnosed developmental disorders in childhood and adolescence. In line with the definition proposed by the American Psychiatric Association’s Diagnostic and Statistical Manual of Mental Disorders [1], an SLD involves difficulties in learning academic skills, such as reading, writing, and mathematics. Generally, the prevalence of SLD is 5–15% among school-age children across different languages and cultures. Specifically, the DSM-5 [1] identifies the existence of four different SLDs:

- *Dyslexia* concerns difficulties in reading skills and, in particular, deficits in accuracy, fluency, and comprehension;
- *Disortography* indicates difficulties in writing abilities, such as lack of encryption processes (phoneme–grapheme conversion), and deficits in written expression, leading to inability to perform written sentences and texts;
- *Dysgraphia* regards difficulties in driving processes of writing skills as a deficit in the graphic design of letters;
- *Dyscalculia* indicates impairment in the reduction in arithmetic and mathematical capabilities and, in particular, in the calculation, enumeration, and memorization of numerical facts.

The causes of SLD are found in neurobiological dysfunctions involved in the normal acquisition process of reading, writing, and calculation. In addition, environmental factors, such as school, family, and social context, are linked with neurobiological ones and help define a greater or lesser adaptation [2].

The main feature of this disorder is that it occurs in individuals with an average intelligence quotient (not less than an IQ equal to 85) and without neurological or sensory deficits, psychopathology, and socio-cultural disadvantages. This characteristic creates a gap between what it expects from a subject with high intellectual abilities in the school environment and its real performance that, in most cases, is poor due to the learning disorder. In this regard, in the Italian context, considering the guidelines proposed by the Consensus Conference [3], two aspects mainly distinguish specific learning disorder from learning disorder (LD): “specificity” and “discrepancy”. Regarding the first element, the altered functionality affects one or more specific domains significantly but is circumscribed, leaving intact the general intellectual function [4,5]. Instead, “discrepancy” is defined as a difference between general intellectual ability (IQ) and academic achievement. A further aspect of SLD is the “chronicity”: this disorder changes with age and environmental demands, manifesting different characteristics during the evolutionary development of those affected [2].

Finally, comorbidity with other psychopathological disorders, such as attention deficit disorder with or without hyperactivity, anxiety, depression, and behavioral disorders, is also common [6,7]. Moreover, individuals may be suffering from more than one specific learning disorder (internal comorbidity, such as dyslexia and disortography) [2].

In Italy, the diagnosis of an SLD is provided following the Ministry of Health and the regional health system guidelines. It is based on the ICD-11 diagnostic classification system, which refers to the category called “Developmental learning disorder” (ICD-11 code: 6A03) and subtypes concerning impairment in reading (6A03.0), impairment in written expression (6A03.1), impairment in mathematics (6A03.2), and other specified impairment of learning (6A03.3) [8]. The diagnosis of SLD must be provided by the local health authority or private professionals (e.g., psychologists, neuropsychiatrists) and subsequently approved by the local health authority.

It is clear that the impairment of these academic skills (reading, writing, and mathematical skills) has a significant negative impact on the life of individuals with SLD, both on a personal level, causing systematic downgrading of academic success or premature school leaving, and on a social level, as a reduction in the development of the person’s social and working potential [9].

Previously, several studies investigated differences between students with and without SLD across multiple domains of functioning and adjustment [10,11,12]. Results show that compared to peers without SLD, students with SLD have lower levels of academic achievement; in addition, academic learning and performance difficulties, clearly persistent and often painful, are typical experiences of many students with SLD [13]. Further research asks whether the individual and social difficulties perceived by subjects with an SLD result from their school failure or are a specific characteristic of the learning disorder [14,15]. In particular, Cosden et al. [14] have studied the perceptions that families and teachers have towards adolescents with a learning disorder, concluding that academic achievement was not the only challenge faced by adolescents; failures depended on both the typical characteristics of the disorder but also on numerous psychological variables, such as lack of ability to relate to peers, low levels of self-esteem and self-understanding. In addition, Vaughn [15], through a longitudinal study lasting five years, has shown that how students perceive themselves with SLD is influenced by academic and social difficulties, especially in the early grades, or by the identification and labelling process.

Initially, the first studies [16,17] conducted with SLD students highlighted the importance of using compensatory tools to help them immediately recover gaps in their learning processes. In this regard, compensation strategies are essential for their academic success, especially in study and performance strategies [17]. Compensation strategies are defined as learning strategies used to “provide ways of compensating for a learning problem” [18]. Therefore, every student with SLD must be enabled to utilize the most suitable compensatory tools for their disorder. The main compensatory tools used by students with SLD are concept maps (a type of graphic organizer used to help students organize and represent knowledge of a subject); speech synthesis (the artificial production of human speech used to help students who struggle with reading); spell checkers (devices used to help students to identify appropriate words and correct spelling errors during the process of writing and proofreading); summaries and forms (to help students to summarize the main concepts of a subject). Furthermore, attendance at specialized after-school programs is also essential for students with SLD to acquire academic skills and develop appropriate social skills [16].

However, although the use of external strategies, such as compensatory tools, is helping to improve the academic performance of students with SLD, the literature highlights how the latter is notably lacking in the use of intrinsic strategies. Students with SLD present deficits in social and emotional abilities, self-regulation, and the development of metacognitive skills. Several studies demonstrate that, compared with peers without SLD, students with SLD have lower socio-emotional competencies [19,20,21] and poor metacognitive knowledge [22,23]. In light of this empirical evidence, the research began to deepen the issues related to all those individual interpersonal and intrapersonal abilities, such as communication, conflict resolution, interpersonal adaptation, self-efficacy, metacognitive skills, and other personal qualities [24,25]. Such skills are commonly defined as “soft skills” and are analyzed as a protective factor for adolescents’ optimal development. For these reasons, this study aimed to explore the associations between interpersonal adaptation, self-efficacy, and metacognitive strategies of adolescents with and without a specific learning disorder.

### 1.1. Interpersonal Adaptation, Self-Efficacy, and Metacognitive Skills

A first important psychological variable to be considered when studying a specific learning disorder is interpersonal adaptation, understood as students’ degree of adaptation despite difficulties. In the literature, interpersonal adaptation is defined as the processes that occur in human relationships when people mutually adapt their behaviors to those of others [26,27]. More specifically, according to Di Nuovo and Magnano [28,29], interpersonal adaptation can be defined as the skills and behaviors the individual needs to possess in order to enter into adaptive and positive relationships with other people and social groups. The presence of difficulties in these areas can lead to uncomfortable situations that, if prolonged in time, can lead to real maladjustment conditions. These “soft skills” consist of different psychological dimensions (passivity, impulsivity, narcissism, worry for self-image, and social stress) that explain the grade of individual difficulties in adapting themselves to interpersonal relationships. The low levels of assertiveness characterize the dimension of passivity, understood as the ability to affirm one’s own needs and to defend one’s ideas without causing suffering to others and avoiding conflicting relationships; impulsivity refers to the tendency to implement dysfunctional reactions in ambiguous situations; worry for self-image is considered in terms of tension deriving from stress caused by others judgment and by lower levels of self-esteem; social stress is identified in the incapacity to manage the social situations, such as speaking in public effectively. In the previous literature, there is no empirical evidence highlighting the relationship between interpersonal adaptation and the diagnosis of SLD. In general, several studies confirm that SLD students report lower social adjustment levels than peers without SLD [12,30,31,32]. Wiener [12] showed that the ability to establish good relationships with peers could foster social adaptation of adolescents, particularly adolescents with an SLD; compared to peers, the latter showed more significant deficits in social skills and social problem-solving. Moreover, the two groups had many differences regarding friendships, experiences of peer victimization, and loneliness. Students with an SLD were less likely to be socially accepted and more likely to be rejected by their peers without an SLD. The author confirms that promoting positive peer relations can reduce the differences in school and social contexts. In addition, Mirzakhany et al. [33] analyzed the assertiveness skills and anxiety of 80 students with a learning disorder and typical peers in schools in Tehran. Results indicate that adolescents with an SLD showed lesser assertiveness skills than typical peers; also, these students have more anxiety than typical peers, proving an inverse relationship between assertiveness and anxiety. Likewise, other studies confirm how assertiveness is generally a deficient social skill in students diagnosed with SLD [34,35] and the importance of specific trainings to promote its appropriate enhancement [36].

Remediating academic deficits remains the primary focus for students with an SLD. Still, recognizing social skill deficits has led to the increased inclusion of social skills training as an adjunct intervention. These programs typically include a comprehensive assortment of skills that cover areas, such as social problem solving, friendship, conversation, planning, and dealing with feelings [31].

Another important aspect to focus on when studying the factors that improve the academic performances of adolescents with SLD is the concept of self-efficacy. According to Bandura’s perspective, self-efficacy is a set of the individual’s beliefs relating to their competence to plan and perform a series of actions to realize desired goals [37]. In addition, self-efficacy plays a decisive role in the individual’s choices, engagement, effort, and persistence [38,39]. Bandura [40] argues that people who show low levels of self-efficacy in completing a specific task will tend to avoid it; conversely, those who express high levels of self-efficacy will feel competent and more inclined to obtain the results set. Generally, an increased perceived sense of self-efficacy is associated with better academic achievements [41,42,43,44,45,46]. These results are evident if we consider students with an SLD [11,47,48,49,50]. Lackaye et al. [11] analyzed the perceived self-efficacy, mood, effort, and hope in a group of 123 adolescents with an SLD and 123 without an SLD. The outcomes showed that students without an SLD reported higher academic and social self-efficacy than peers with an SLD. In addition, students without an SLD reported higher hope and high effort in their academic tasks than adolescents with SLD. Similar empirical evidence is found in the research conducted by Klassen [48,49,50] that studied two different types of self-efficacy (self-efficacy for self-regulated learning and general self-efficacy) in relation to the spelling and writing efficacy beliefs of adolescents with and without a learning disorder. The results demonstrated that the former over-estimated their spelling and writing performances, whereas the students without an SLD were generally accurate in their performance estimates. Therefore, adolescents with SLD have less awareness of personal abilities to carry out specific academic tasks effectively.

Further studies confirm how high levels of academic self-efficacy are linked to low levels of anxiety, stress, and illness; in particular, Hen and Goroshit [51] examined, in a sample of 287 students with and without an SLD, the differences in academic difficulties and maladaptive academic behaviors. Results indicate that adolescents with an SLD exhibit high levels of learned helplessness, including diminished persistence, lower academic expectations, and negative affect. Such empirical evidence confirms that self-efficacy plays a protective role in the adaptation of students in the school context, especially in the presence of a specific learning disorder.

To conclude the review of studies that mainly highlighted the positive role of interpersonal adaptation and self-efficacy on the quality of life of adolescents with an SLD, we cannot overlook the concept of metacognition. Metacognition was extensively studied in cognitive psychology and defined by Flavell [52] as “knowledge and cognition about cognitive phenomena and monitoring of one’s memory, comprehension, and other cognitive processes” (p. 906). According to this perspective, Senemoğlu [53] considered metacognition as “knowledge of one’s cognitive system, structure, and functioning; in other words, the awareness of one’s cognitive structure and the learning characteristics and the ability to monitor and regulate one’s cognitive processes”. (p. 336). Starting from these definitions, it is possible to distinguish two aspects of this psychological construct: metacognitive knowledge and metacognitive experiences. The first is the knowledge about general strategies that can be used for completing a specific task, awareness of their use in certain situations, and the ability to identify those learning units where such a strategy can be most effective. The second consists of monitoring, controlling, and regulating cognition. Several studies showed that adequate metacognitive skills positively affect students’ learning abilities [54,55,56,57,58,59]. This is even more evident in the case of students with poor academic performance due to a learning disorder, especially in mathematics, reading, and comprehension [54,60,61,62]. More specifically, it has been shown that students with a learning disorder cannot spontaneously transfer the strategies they learned from previous contexts to new contexts [17].

For this reason, these students must develop the ability to understand why and when to apply these skills and monitor their implementation. Additionally, several studies underlined the importance of teaching metacognitive strategies to make students with an SLD reach academic success and increase their motivation and self-efficacy [17]. For example, in a recent study, Wang et al. [63] showed that, in a sample of students with a LD, high metacognition levels and correct use of metacognitive strategies correlated negatively with academic failures and positively with self-efficacy. Furthermore, the recent literature reports several studies, demonstrating the effectiveness of the interventions, promoting metacognitive strategies to improve LD students’ academic performances [22,64,65].

### 1.2. Purpose of Study and Hypotheses

This study aimed to explore the association of interpersonal adaptation with generalized self-efficacy and metacognitive skills expressed by secondary school and high school students with and without an SLD. Considering the previous literature on the relationships between the SLD and the psychological variables analyzed, we formulated the following study hypotheses:

o H1: adolescents with an SLD report lower interpersonal adaptation than adolescents without an SLD;
o H2: adolescents with an SLD show lower general self-efficacy than adolescents without an SLD;
o H3: adolescents with an SLD report lower metacognitive skills than adolescents without an SLD;
o H4: adolescents with an SLD using compensatory tools express high interpersonal skills, self-efficacy, and metacognitive skills than adolescents with an SLD not using compensatory tools;
o H5 MAIN OBJECTIVE: the use of compensatory tools is predictive of high levels of metacognitive skills in adolescents with an SLD (H5).

**Figure 1:**
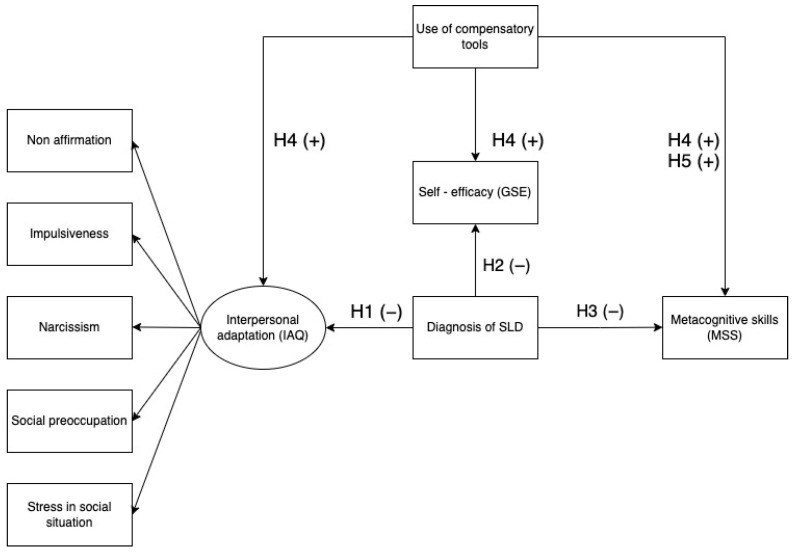
Conceptual framework and hypotheses of the study.

Figure 1 summarizes the conceptual framework of this study. A plus sign (+) indicates a positive relationship, while a minus sign (−) indicates a negative relationship between the variables.

## 2. Materials and Methods

### 2.1. Study Design and Participants

This is an observational, prospective study performed at the Department of Educational Sciences of the University of Catania, aiming to explore the association of interpersonal adaptation with generalized self-efficacy and metacognitive skills expressed by secondary school and high school students with and without an SLD. Eligible participants attended lower and upper secondary schools in the province of Catania who were invited to give their willingness to participate in the study. The students with SLD were recruited from specialized centers operating in the Catania city area that already provided diagnoses for all the participants, following the standard ICD-11 criteria [8].

The authors respected the Ethical Code for Italian psychologists (L. 18.02.1989, n. 56), the Legislative Decree for the privacy of provided data (DLGS 196/2003), and the Ethical Code for Psychological Research (27 March 2015) established by the Italian Psychologists Association. No sensitive data that could identify the participants was collected. The schools involved in the research had previously informed the students’ parents to consent to the study’s participation. The Internal Ethics Review Board (IERB) of the Department of Educational Sciences approved the study.

### 2.2. Measures

#### 2.2.1. Socio-Demographic Questionnaire

A socio-demographic questionnaire was administered to the participants to collect information on gender, age, nationality of parents, type of school attended, and regularity of school career. Furthermore, students with SLD were asked to indicate their specific diagnoses (dyslexia, dyscalculia, disortography, dysgraphia), use or non-use of compensatory tools, and tools they used.

#### 2.2.2. Interpersonal Adaptation Questionnaire (IAQ)

The IAQ [28,29] is a self-report inventory with a set of items for each of which students evaluate themselves on a 3-points frequency scale from 0 to 2 points (“0 = never; 1 = sometimes; 2 = often”). Specifically, the IAQ is composed of 75 sentences, grouped into the following five subscales: *Non-affirmation* (“I’m able to defend my rights-reverse item”; “I honestly express my feelings to everyone-reverse item”); *Impulsiveness* (“People think that it’s too difficult to agree with me”; “It sometimes happens that I insulted someone”); *Narcissism* (“I like to be in others’ attention”; “I like my physical appearance”); *Social preoccupation* (“When I am with other people, I’m worried I’m behaving in a ridiculous way”; “I’m afraid that other people could refuse what I do”); and *Stress in social situations* (“When I talk to others for the first time, I feel myself worried”; “Speaking in public is a problem to me”). In this study, the internal consistency of all the subscales of IAQ is satisfactory (from 83 to 93).

#### 2.2.3. Generalized Self-Efficacy Scale (GSE)

The GSE [66] assesses the general sense of perceived self-efficacy to predict dealing with critical daily events and adaptation after experiencing stressful life situations. This scale is composed of 10 items (“I can always manage to solve difficult problems if I try hard enough”, “I can remain calm when facing difficulties because I can rely on my coping abilities”, “If I am in trouble, I can usually think of a solution”) on a Likert scale ranging from 1 (corresponding to “not at all true”) to 4 intervals (corresponding to “exactly true”). The total score ranges from 10 to 40 points. For this scale, the internal consistency is satisfied for our sample with a value of α equal to 93.

#### 2.2.4. Metacognitive Skills Scale (MSS)

The MSS [67] was used to assess the metacognitive skills and consists of 30 items with responses on a 5-point Likert scale ranging from 1 (corresponding to “strongly disagree”) to 5 intervals (corresponding to “strongly agree”). Overall, this tool evaluates how individuals tend to use their metacognitive knowledge and learning strategies (e.g., “I use my previous experiences while organizing my new learnings”, “I assess if the cognitive strategy that I employ has been successful or not”); how much they are aware of their learning abilities (e.g., “I don’t have an exact idea of how to organize my learning-reverse item”, “I prepare the learning environment that is necessary for learning process”); and how the individuals can plan, monitor, and modify the strategies used to make learning more effective (e.g., “It is important for me to overview my learnings from time to time to determine how much and what I learned”, “I don’t spare much time for monitoring how much I learned about the subject during learning process-reverse item”). The total score ranges from 55 to 275 points. The internal consistency of MSS for this study is good, with a value of α equal to 94.

### 2.3. Procedure

The study participants were tested during and after school hours in individual and group settings (max. five subjects) between November 2019 and February 2020. Two psychologists administered the questionnaires.

The study procedures were explained to the students, and any questions they had were answered. The instructions stated that the questionnaires were voluntary and that the responses were confidential. The completion of the questionnaires took about 45 min.

### 2.4. Statistics

The sample has been described in its clinical and demographic characteristics using descriptive statistics techniques. Qualitative variables have been described with absolute frequencies and percentages, and quantitative variables have been summarized with mean and standard deviation. Comparisons between the two groups of patients have been performed by applying the chi-square test (or the Fisher exact test) for categorical variables. The Mann–Whitney test has been used for continuous, not normally distributed variables. To evaluate hypothesis H5, which is the main objective of the study, logistic regression has been performed, considering the dichotomized version of MSS TOT (MSS TOT has been divided into high and low assuming the median as cut-off) as a dependent variable and the variable taking value = 0 in case of no compensatory tools used and value = 1 if at least one compensatory tool is in use as an independent one. Effect size has been calculated and addressed as well. A *p*-value < 0.05 has been considered significant. Analyses were performed using the Statistical Package for the Social Sciences (SPSS) version 25.0 (IBM Corporation, Armonk, NY, USA).

## 3. Results

A total of N = 564 subjects, 41.8% males and 58.2% females, have been included in the study. The mean age of the general sample is 16.1 years (SD = 1.7). In the sample, 175 students were diagnosed with SLD (31.0%). Table 1 reports demographic details about students’ two study groups (SLD and NO-SLD).

The sample size is appropriate to investigate the study’s primary objective (H5). In more detail, according to the rule of thumb [68], a total number of 10 events are required to test one single predictive factor. In this study, there are 277 students with a high level of metacognitive skills, and there is only one predictive factor (the use of compensatory tools).

The distribution of sex in the groups presents a statistically significant difference (*p* = 0.007), with 61.7% and 50.3% females in the NO-SLD group and the SLD group, respectively. The mean age was 16.6 years (SD = 1.05) in the NO-SLD group and 15.1 years (SD = 2.3) in the SLD group. This difference is statistically significant (*p* < 0.001).

One hundred twenty-seven attended the lower secondary school, 312 the lyceum, and 125 a professional school. Most students in both the groups (94.1% in the NO-SLD group and 80.6% in the SLD group) present a regular school career. However, the difference in the proportions is significant (*p* < 0005).

Regarding the SLD diagnoses, 44.6% of SLD students were affected by dyslexia, 32.6% presented dyscalculia, 34.3% disortography, and 26.9% dysgraphia.

About compensatory tools, these instruments have been used by 78.9% of students in the SLD group. Within the SLD group, 21.1% of students claimed to use three different types of compensatory tools.

Concept maps are the most used tool (57.7%), followed by speech synthesis (26.9%), summaries (25.7%), spell-checkers (22.9), and forms (1.1%). Furthermore, 48% of the subjects attended a specialized after-school program.

Table 2 shows the characteristics of the students with SLD in our sample.

Concerning hypothesis H1 (adolescents with an SLD report lower interpersonal adaptation than adolescents without an SLD), Table 3 shows that SLD students present all the scores with higher values than NO-SLD students. The differences are significant, although the effect sizes are all extremely small.

Concerning hypothesis H2 (adolescents with an SLD show lower general self-efficacy than adolescents without an SLD), Table 4 shows that SLD students present a lower value of the GSE TOT score than NO-SLD students. However, the difference is not significant (*p* = 0.07). Moreover, the effect size is extremely small.

Concerning hypothesis H3 (adolescents with an SLD report lower metacognitive skills than adolescents without an SLD), Table 5 shows that SLD students present MSS TOT values higher than NO-SLD students. The difference is significant (*p* < 0.001), but the effect size is extremely small.

Concerning the hypothesis H4 (adolescents with an SLD using compensatory tools express higher interpersonal skills, self-efficacy, and metacognitive skills than adolescents with an SLD not using compensatory tools), Table 6 shows that SLD students using compensatory tools present higher MSS TOT values than SLD students not using compensatory tools (121.33 vs. 86.57, respectively). The differences are all statistically significant (*p* < 0.001), but the effect sizes are small.

Finally, concerning H5 (the use of compensatory tools is predictive of high levels of metacognitive skills in adolescents with an SLD), the logistic regression analysis highlighted that using at least one compensatory tool increases the odds of presenting a high level of metacognitive skills (MSSTOT > median = 99) by 14.140. In more detail, the odds of presenting a high level of metacognitive skills are 14.140 times greater for students using at least one compensatory tool as opposed to those not using a compensatory tool at all (OR = 14.140, 95% CI = [5.15, 38.7], *p* < 0.001).

## 4. Discussion

This study compared a sample of Italian secondary school and high school students with an SLD to a control group without an SLD. The first aim was to explore the differences in interpersonal adaptation, self-efficacy, and metacognition between students with and without an SLD. As a second aim, we investigated the relationships between these variables and the predictors of self-efficacy and metacognition levels in the general sample and the sub-group of students with an SLD.

The results confirmed our hypothesis (H1) that students with an SLD reported a lower interpersonal adaptation than students without an SLD. More specifically, in this study, students with an SLD showed high levels of non-affirmation, so they had difficulty asserting their needs and ideas without conflicts with others. We also found a significant interaction between the effects of a diagnosis of an SLD and the type of school on non-affirmation. Considering that minimal literature data exist on the relationship between interpersonal adaptation and SLD, our data provide preliminary evidence to confirm that adolescents with an SLD showed more significant social and interpersonal skills deficits. In particular, assertiveness seems to be the most impaired interpersonal skill in adolescents with an SLD, as already highlighted in the study by Mirzakhany et al. [33]. This finding is also confirmed by other studies [34,35], showing that a low level of assertiveness in students with SLD is a significant indicator of problems with peers. In this regard, assertiveness training for students with SLD effectively improves social and interpersonal skills and is recommended to prevent maladaptive behavior and relationship difficulties [36].

Our findings are consistent with previous results showing that students with an SLD are more impulsive and have more problems handling social situations and relationships with others [32,34,35]. Therefore, these data confirm that learning disorders also significantly impact the social and relational skills of adolescents suffering from them, as highlighted in the literature on the topic [12,30,31]. Therefore, future research will have to clarify better how a diagnosis of SLD affects the so-called “soft skills” and interpersonal skills in adolescence.

The results also confirm the second hypothesis of this study. Indeed, students with an SLD in the sample reported significantly lower levels of self-efficacy than their peers without an SLD. These findings support our hypothesis based on previous studies that the presence of a learning disorder is associated with a decrease in the adolescent’s awareness and confidence in their ability to complete a task and achieve a goal [11,47,48,49,50].

Contrary to our hypothesis (H3), students with an SLD in our sample reported higher metacognition scores than peers without an SLD. This result is certainly worthy of discussion as the literature on the topic points out that students with an SLD generally have poorer metacognitive skills, leading to difficulties in transferring the strategies they learned from previous contexts to new contexts [17]. To confirm this result, Trainin and Swanson [65] indicated that students with an SLD benefit more from high metacognitive strategies than peers without an SLD. In fact, in this case, metacognitive strategies are linked to more efficient ways to improve performance in academic contexts. However, it was also found that students using compensatory tools reported higher levels of metacognition than students who did not use these compensatory tools (H4). In addition, logistic regression analysis showed that the odds of presenting a high level of metacognitive skills are 14.140 times greater for students using at least one compensatory tool than those not using a compensatory tool (H5). These findings confirm the importance of using appropriate compensation measures to improve the self-regulation strategies of students with SLD.

Furthermore, the use of compensatory tools was also associated with better interpersonal skills and higher levels of self-efficacy in students with SLD (H4), confirming the fundamental importance of these instruments in promoting and improving not only learning but also the personal and social skills of students with SLD.

This study confirms that a diagnosis of SLD is a risk factor for worse personal and social functioning. Still, the use of appropriate compensation strategies and specific training for developing and enhancing skills, such as interpersonal skills or self-efficacy, can be an important protective factor against the negative impact of learning difficulties on students’ personal and social functioning and the quality of their school experience.

Some limitations of this study need to be considered. First, the cross-sectional design of our study cannot prove causation because all the variables cannot be determined. Secondly, self-report questionnaires were used in this study; thus, the reliability of the responses and the consequent self-report bias cannot be proved. Finally, other important variables that are significantly associated with the psychological well-being of students with an SLD were not included in this study, such as self-concept and locus of control, resilience, hope and investment of effort, and self-esteem.

## 5. Conclusions

In conclusion, our results suggest that the presence of a diagnosis of an SLD is significantly associated with negative self-efficacy, together with reduced interpersonal skills. However, further studies are needed on the psychological well-being of adolescents with an SLD and especially on the protective role of personal, social, and environmental characteristics.

Currently, in Italy, those who receive a diagnosis of SLD is protected by Law 170 (2010), which aims to protect students with SLD and their right to education and to promote their success at school. In this sense, studying like other students means that students with SLD have the right to access “educational support measures and compensatory tools” that allow them to express their potential. Specifically, four fundamental points will enable the realization of an effective teaching intervention oriented to the academic success of students with SLD: (1) the early identification and enhancement through a screening process with individual tests in the affected areas (reading, writing, calculation), which allows the placement of pupils in a performance range that can signal the need to start the enhancement. It consists of activities to stimulate the skills in which the student has difficulties; (2) the individualized and personalized teaching that refers to individual recovery activities, in class or in a specialized after-school program, that help the student to enhance skills and acquire or improve their skills; (3) the personalized teaching plan that consists of an educational project in which the steps and tools necessary to foster student learning and academic success with DSA are indicated; (4) the compensatory instruments and relief measures, which replaces or facilitates the performance required in the deficit ability, be it writing, reading, or calculation (e.g., speech synthesis, computers, tablets, word processors with spell checker, calculator, concept maps, oral or digital tests instead of written tests, to avoid reading aloud, not having to perform time trials or having more time available than peers, avoid surprise questions by programming them).

This study confirms the effectiveness of these measures in supporting students with SLD, as well as the need to implement specific programs to enhance soft skills in students with SLD and an in-depth assessment of their effectiveness in improving other skills not explicitly investigated in this study, such as self-esteem and resilience.

## Figures and Tables

**Table 1:** Sociodemographic characteristics of the sample.

		NO SLD (N = 389)	SLD (N = 175)	TOT (N = 564)
Sex	Male	38.3%	49.7%	41.8%
	Female	61.7%	50.3%	58.2%
Nationality of parents	Italian	94.9%	91.4%	93.8%
	Not Italian	5.1%	8.5%	6.2%
Attended school	Lower secondary school	17.0%	34.9%	22.5%
	Higher secondary school	63.0%	38.3%	55.3%
	Professional school	20.1%	26.9%	22.2%
Scholastic career	Regular	94.1%	80.6%	89.9%
	Not regular	5.9%	19.4%	10.1%

**Table 2:** Characteristics of the group of students with SLD.

		SLD (N = 175)
Dyslexia	No	55.4%
	Yes	44.6%
Dyscalculia	No	67.4%
	Yes	32.6%
Disortography	No	65.7%
	Yes	34.3%
Dysgraphia	No	73.1%
	Yes	26.9%
Compensatory tools used (number of)	0	21.1%
	1	20.6%
	2	20.6%
	3	21.1%
	4	14.3%
	5	1.7%
	6	0.6%
Conceptual maps	No	42.3%
	Yes	57.7%
Summaries	No	74.3%
	Yes	25.7%
Speech synthesis	No	73.1%
	Yes	26.9%
Spell checker	No	77.1%
	Yes	22.9%
Forms	No	86.9%
	Yes	13.1%
Specialized after-school	No	52.0%
	Yes	48.0%
Self-instruction techniques	No	90.9%
	Yes	9.1%
Calculus rehabilitation	No	93.7%
	Yes	6.3%
Attention rehabilitation	No	92.0%
	Yes	8.0%
Reading speed rehabilitation (tachistoscopic)	No	93.1%
	Yes	6.9%
Praxia rehabilitation	No	92.6%
	Yes	7.4%
Memory exercises	No	92.0%
	Yes	8.0%
Reading and comprehension rehabilitation	No	93.1%
	Yes	6.9%
Eye-manual coordination	No	95.4%
	Yes	4.6%
Spelling rehabilitation	No	94.3%
	Yes	5.7%
Work on muscle tone	No	96.6%
	Yes	3.4%
Metaphonological skills	No	93.1%
	Yes	6.9%
Lexical inference techniques	No	94.9%
	Yes	5.1%
Graphemes–phonemes conversion	No	94.9%
	Yes	5.1%
Spelling and oral string conversion	No	96.6%
	Yes	3.4%
Repeated readings with facilitations	No	94.9%
	Yes	5.1%
Semantic inference techniques	No	96.6%
	Yes	3.4%
Lexical enhancement interventions	No	94.9%
	Yes	5.1%
Metacognitive strategies	No	94.3%
	Yes	5.7%
Psychotherapy	No	89.1%
	Yes	10.9%

**Table 3:** Differences in interpersonal skills scores in students with and without SLD.

	NO-SLD		SLD		*p* *	*η* ^2^
	*M*	*SD*	*M*	*SD*		
IAQ-A	0.71	0.30	1.00	0.54	<0.001	0.08
IAQ-I	0.77	0.38	0.94	0.46	<0.001	0.05
IAQ-N	0.86	0.29	0.94	0.53	0.021	0.01
IAQ-P	0.96	0.38	1.01	0.33	0.035	0.01
IAQ-S	0.64	0.41	0.97	0.58	<0.001	0.07

Notes: SLD = specific learning disorder; IAQ-A = interpersonal adaptation questionnaire—non affirmation; IAQ-I = interpersonal adaptation questionnaire—impulsiveness; IAQ-N = interpersonal adaptation questionnaire—narcissism; IAQ-P = interpersonal adaptation questionnaire—social preoccupation; IAQ-S = interpersonal adaptation questionnaire—stress in social situation. * Mann–Whitney U test.

**Table 4:** Differences in self-efficacy scores in students with and without SLD.

	NO-SLD		SLD		*p* *	*η* ^2^
	*M*	*SD*	*M*	*SD*		
GSE TOT	28.52	4.95	25.90	10.18	0.07	0.01

Notes: SLD = specific learning disorder; GSE = general self-efficacy scale. * Mann–Whitney U test.

**Table 5:** Differences in metacognition scores in students with and without SLD.

	NO-SLD		SLD		*p* *	*η* ^2^
	*M*	*SD*	*M*	*SD*		
MSS TOT	98.77	18.20	113.98	30.42	<0.001	0.04

Notes: SLD = specific learning disorder; MSS = metacognitive skills scale. * Mann–Whitney U test.

**Table 6:** Differences in interpersonal skills, self-efficacy, and metacognition scores in students with SLD according to the use of compensatory tools.

	SLD Students Not Using Compensatory Tools		SLD Students Using Compensatory Tools		*p* *	*η* ^2^
	*M*	*SD*	*M*	*SD*		
IAQ-A	1.40	0.41	0.88	0.51	<0.001	0.1
IAQ-I	0.77	0.49	0.97	0.44	<0.038	0.02
IAQ-N	0.55	0.46	1.03	0.50	<0.001	0.1
IAQ-P	1.21	0.28	0.95	0.32	<0.001	0.1
IAQ-S	1.49	0.38	0.83	0.55	<0.001	0.2
GSE TOT	17.03	6.30	28.28	9.70	<0.001	0.2
MSS TOT	86.57	12.98	121.33	29.55	<0.001	0.04

Notes: SLD = specific learning disorder; IAQ-A = interpersonal adaptation questionnaire—non affirmation; IAQ-I = interpersonal adaptation questionnaire—impulsiveness; IAQ-N = interpersonal adaptation questionnaire—narcissism; IAQ-P = interpersonal adaptation questionnaire—social preoccupation; IAQ-S = interpersonal adaptation questionnaire—stress in social situation; GSE = general self-efficacy scale; MSS = metacognitive skills scale. * Mann–Whitney U test.

## Data Availability

The data presented in this study are available on request from the corresponding author. The data are not publicly available due to department policy.
